# Effect of new year holidays on hospital mortality: a time series study

**DOI:** 10.1186/s12245-019-0243-x

**Published:** 2019-08-27

**Authors:** Mohadeseh Ghanbari Jahromi, Reza Goudarzi, Vahid Yazdi-Feyzabadi, Saeed Amini, Javad Nazari, Mohammadreza Amiresmaili

**Affiliations:** 1 Department of Health Management, Policy and Economics, School of Management and Medical Informatics, Kerman University of Medical Sciences, Kerman, Iran; 2 Modeling in Health Research Center, Institute for Futures Studies in Health, Kerman University of Medical Sciences, Kerman, Iran; 3 Health Services Management Research Center, Institute for Futures Studies in Health, Kerman University of Medical Sciences, Kerman, Iran; 4 Department of Health Services Management, School of Health, Arak University of Medical Sciences, Arak, Iran; 5 Department of Pediatrics, School of Medicine, Arak University of Medical Sciences, Arak, Iran

**Keywords:** Nowruz holidays, Hospital mortality, Time series, Iran

## Abstract

**Background:**

Nowruz holidays, as one of the most important holidays in Iran, can lead to changes in the trend of hospital deaths. Due to changes in lifestyle and increased accidents, hospitals become crowded during the holidays. The present study aimed to investigate the effect of Nowruz holidays on hospital deaths at teaching hospitals affiliated with the Kerman University of Medical Sciences in southeast Iran.

**Methods:**

The research population included all hospital deaths during the period from 23 August 2013 to 21 September 2016. Data on hospital deaths, including age, sex, work shift, cause of death and ward type were collected daily from the Hospital Information System. Data were analysed using *t* test and time series regression models, in Stata 13.0.

**Results:**

The holiday deaths primarily occurred in males (57.14%) and people aged 60–79 years (29.20%). More than half of the holiday deaths occurred in the morning shift (59.88%). The leading cause of holiday deaths was injuries, poisoning and other consequences of external causes (25.31%). Most holiday deaths occurred in the ICU (53.88%). Death rate per day during the Nowruz holidays was higher than it was during working days and non-Nowruz holidays (1.36%).

**Conclusions:**

Reduced quality of services during the holidays is a prominent issue and leads to increased hospital death. Hospital managers can improve the quality of services, by identifying the root causes and by taking measures such as increased and balanced distribution of human resources, equipping hospitals and improving supervision during holidays.

## Background

Abbas Kiarostami, a prominent Iranian artist and winner of the Golden Palm Award of the Cannes Film Festival in 2012, had surgery due to intestinal disease just before Nowruz holidays. His condition worsened during the holidays and he died on July 4, 2016. Following this event, many discussions arose on social media, including Telegram, Facebook, Twitter and news websites [1, 2]. This event highlighted the poor quality of hospital services during the Nowruz holidays.

Nowruz is the name of the Iranian New Year, also known as the Persian New Year, which is celebrated worldwide by Iranians, along with some other ethno-linguistic groups, as the beginning of the New Year. Nowruz is the day of the vernal equinox and marks the beginning of spring in the Northern Hemisphere. It marks the first day of the first month (Farvardin) in the Iranian calendar. It usually occurs on March 21 or the previous or following day, depending on where it is observed. The moment the sun crosses the celestial equator and equalizes night and day, families gather together to hold the rituals.

Nowruz holiday in Iran informally is a 2-week event. Although on the basis of the official Iranian’s solar calendar the holidays for civil servants and public workers formally is 4 days, the public holidays during new Persian year continues till 2 weeks (i.e. 14 days). On the other hand, every worker (all those working in government/public sector) based on employment law has 30 days as earned leaves or privilege leaves (vacation) which mostly workers decide to use it after the formal 4-day holidays in the new Persian year known as Nowruz holidays. This long-term holiday may affect the performance of various sectors, including education and health care [3]. Hospitals, as a healthcare institution, cannot remain ineffective during these holidays. Holidays can also lower the quality of hospital services and endanger the health of patients. Additionally, the Nowruz holidays change the behaviour of individuals, often resulting in more accidents. During holidays, most hospitals are faced with shortages of medical personnel [4], emergency congestion [5–7] and postponement of treatment processes [8].

In these holidays, the pattern of hospitalization is largely affected by several factors. One of the important factors in this regard is hospitalization due to the increase in the number of road traffic accidents [9]. The consumption of alcohol and changes in diet are among issues that are also associated with holiday deaths [10]. Many people harm themselves by consuming unhealthy fatty foods [11] resulting in weight gain [12]. Many people are also faced with stress on how to deal with relatives’ visits, economic pressures, house decoration and travel plans during these days [10]. Thus, all of these factors can potentially lead to changes in hospital mortality rate.

Various studies have been conducted on the deaths during holidays. An Iranian study showed that death rate has increased during the holidays and subsequent days [13]. Another study in the UK showed that the death rate increases during the Christmas holidays and the number of healthcare staff decreases due to holiday leave [4]. A study in Canada also indicated that holidays are a dangerous period for patients with stroke [14]. Various studies have shown that mortality of cardiac patients is higher during New Year holidays [15, 16]. Other studies have shown that deaths during holidays in emergency departments [17] and critical care units [18] are more frequent than during normal working days. Bergen and Hawton in their study concluded that the rate of suicide attempts during Christmas and New Year increases [19]. Moreover, demand for hospital services for cardiovascular, respiratory and trauma patients increases during holidays [20]. A study showed that the death rate in educational hospitals is higher than non-teaching hospitals [21].

Hospital mortality, which refers to deaths occurring within 24 h after hospitalization [22], is one of the important factors in assessing the quality of hospital services, which account for a large proportion of deaths. It is closely linked to hospitals’ facilities, equipment, quality of medical and nursing services, type of hospital services and demographics (age, gender, and economic status) [23]. Furthermore, it seems that different factors may affect the hospital mortalities which demand further studies on this issue. Despite some previous studies on holiday deaths, there is not enough evidence and literature on the effect of holidays on hospital mortalities. The aim of this study was to investigate the rate and trend of hospital mortality during Nowruz holidays in teaching hospitals of Kerman, southeast of Iran.

## Methods

### Variables and data collection

In this retrospective study, the research population included all teaching hospitals affiliated with the Kerman University of Medical Sciences. The data about demographic status, work shifts, cause of death and wards were obtained from the Hospital Information System (HIS), from three teaching hospitals, labelled A, B and C, between August 23, 2013, and September 21, 2016. The causes of death were classified according to the main codes in the ICD 10, which included 22 disease groups. Work shifts were classified as either morning, evening or night shifts. In this study, the Nowruz holiday was considered to be March 16–April 3.

### Model development

Data were entered into the Stata software version 13 and were analysed using descriptive statistics (relative frequency), independent *t* test and time series regression model, and for models, the coefficient of determination (*R*^2^) and Dickey-Fuller test were used.

Equation:

$$ {y}_t={a}_0+{a}_1{x}_{1t}+\dots +{a}_n{x}_{it} $$

In the regression model used in this study, “*y*” was the dependent binomial variable (hospital death), “*t*” was the day, *a*_0_ was the vertical intercept, *a*_1_ was the independent variable coefficient and *x*_1_ was the independent variable (virtual variable of hospital mortality during the Nowruz holidays as number zero and the variable of normal working days as the number one)*.* The percentage of hospital deaths was obtained by the number of hospital deaths per day divided by the total number of hospitalizations per day.

## Results

The total number of hospitalizations, mean age of the patients and number of deaths in the three educational hospitals during the 3-year period of the study were 320,347, 47.34 and 5414, respectively. Men (58.66% of deaths) and morning shift (63.42% of deaths) accounted for the highest mortality rates. The most common cause of death (21.77%) was due to injuries, poisoning and other consequences of external causes. A majority of deaths occurred in the ICU (58%). From 5414 deaths, 245 deaths (1.69%) occurred during Nowruz holidays, and the rest occurred during the non-holiday periods. Among these 245 deaths, 140 deaths (57.14%) occurred in the 60–79-year-old males.

Furthermore, 103 deaths (59.89%) occurred in morning shifts during the Nowruz holiday. The most common cause of holiday deaths (25.30%) was due to injuries, poisoning or other consequences of external causes. Holiday deaths mostly occurred in the ICU (53.88%). Other information related to the research variables is presented in Table 1.

**Table 1:** Demographic characteristics of hospital deaths according to Nowruz holidays and Non-Nowruz Holiday days from 23 August 2013 to 21 September 2016

Indicator	Nowruz holiday frequency (%)	Non-Nowruz holiday frequency (%)	Total
Sex			
Male	140 (57.14)	3041 (58.83)	3181 (58.75)
Female	105 (42.86)	2128 (41.17)	2233 (41.25)
Total	245 (100)	5169 (100)	5414 (100)
Shift			
Morning	103 (59.89)	2375 (63.59)	2478 (63.42)
Evening	32 (18.60)	729 (19.52)	761 (19.48)
Night	37 (21.51)	631 (16.89)	668 (17.10)
Total	172 (100)	3735 (100)	3907 (100)
Code of death			
A00–B99 (certain infectious and parasitic diseases)	10 (4.08)	257 (4.97)	267 (4.93)
C00–D48 (neoplasms)	33 (13.47)	717 (13.87)	750 (13.85)
D50–D89 (diseases of the blood and blood-forming organs and certain disorders involving the immune mechanism)	2 (0.81)	57 (1.10)	59 (1.09)
E00–E90 (endocrine, nutritional and metabolic diseases)	1 (0.41)	63 (1.22)	64 (1.18)
F00–F99 (mental and behavioural disorders)	0	0	0
G00–G99 (diseases of the nervous system)	3 (1.22)	97 (1.88)	100 (1.85)
H00–H59 (diseases of the eye and adnexa)	0	0	0
H60–H95 (diseases of the ear and mastoid process)	0	0	0
I00–I99 (diseases of the circulatory system)	28 (11.43)	745 (14.41)	773 (14.28)
J00–J99 (diseases of the respiratory system)	32 (13.06)	632 (12.23)	664 (12.26)
K00–K93 (diseases of the digestive system)	8 (3.27)	275 (5.32)	283 (5.23)
L00–L99 (diseases of the skin and subcutaneous tissue)	0	9 (0.17)	9 (0.17)
M00–M99 (diseases of the musculoskeletal system and connective tissue)	0	19 (0.37)	19 (0.35)
N00–N99 (diseases of the genitourinary system)	5 (2.04)	155 (3)	160 (2.95)
O00–O99 (pregnancy, childbirth and the puerperium)	0	4 (0.08)	4 (0.07)
P00–P96 (certain conditions originating in the perinatal period)	33 (13.47)	603 (11.67)	636 (11.75)
Q00–Q99 (congenital malformations, deformations and chromosomal abnormalities)	9 (3.67)	215 (4.16)	224 (4.14)
R00–R99 (symptoms, signs and abnormal clinical and laboratory findings, not elsewhere classified)	19 (7.76)	204 (3.94)	223 (4.12)
S00–T98 (injury, poisoning and certain other consequences of external causes)	62 (25.30)	1114 (21.55)	1176 (21.72)
V01–Y98 (external causes of morbidity and mortality)	0	3 (0.06)	3 (0.06)
Z00–Z99 (factors influencing health status and contact with health services)	0	0	0
U00–U99 (codes for special purposes)	0	0	0
Total	245 (100)	5169 (100)	5414 (100)
Ward			
Internal	90 (36.73)	1738 (33.63)	1828 (33.77)
ER	17 (6.94)	301 (5.82)	318 (5.87)
Surgery	6 (2.45)	122 (2.36)	128 (2.36)
ICU	132 (53.88)	3008 (58.19)	3140 (58)
Total	245 (100)	5169 (100)	5414 (100)

On 23 Aug 2013–20 Mar 2014, the percentage of hospital deaths during non-holiday periods was greater than that of Nowruz holidays. But in general, the proportion of hospital deaths during the Nowruz holidays was 2.38% per hospital stay per day from August 23, 2013, to September 21, 2016, which was 1.36 times higher than the non-holiday periods (Table 2).

**Table 2:** Comparison of hospital death rate for hospitalization per day on holidays and non-holidays in Kerman educational hospitals from 23 August 2013 to 21 September 2016

Hospitals	Year	Percentage of death to total admission		Total	*T*	*P* value
		Holidays	Non-holidays			
A	23 Aug 2013–20 Mar 2014	3.41	1.98	2.02	1.90	0.05
	21 Mar 2014–20 Mar 2015	3.14	2.33	2.37	1.76	0.07
	21 Mar 2015–19 Mar 2016	2.82	2.78	2.78	0.09	0.92
	20 Mar 2016–21 Sep 2016	4	3.06	3.12	1.56	0.11
	23 Aug 2013–21 Sep 2016	3.26	2.53	2.56	2.73	0.006
B	23 Aug 2013–20 Mar 2014	1.31	1.47	1.46	− 0.25	0.79
	21 Mar 2014–20 Mar 2015	2.16	1.36	1.40	2.71	0.006
	21 Mar 2015–19 Mar 2016	1.45	1.08	1.09	1.41	0.15
	20 Mar 2016–21 Sep 2016	1.46	1	1.03	1.29	0.19
	23 Aug 2013–21 Sep 2016	1.68	1.23	1.25	2.62	0.008
C	23 Aug 2013–20 Mar 2014	0.68	1.53	1.51	− 0.90	0.36
	21 Mar 2014–20 Mar 2015	3.2	1.39	1.48	3.74	0.0002
	21 Mar 2015–19 Mar 2016	1.72	1.59	1.59	0.25	0.79
	20 Mar 2016–21 Sep 2016	2.08	1.40	1.45	1.25	0.21
	23 Aug 2013–21 Sep 2016	2.20	1.48	1.52	2.51	0.01
Total	23 Aug 2013–20 Mar 2014	1.80	1.66	1.66	0.31	0.75
	21 Mar 2014–20 Mar 2015	2.83	1.69	1.75	4.55	0.000
	21 Mar 2015–19 Mar 2016	2	1.81	1.82	0.68	0.49
	20 Mar 2016–21 Sep 2016	2.51	1.82	1.87	2.10	0.03
	23 Aug 2013–21 Sep 2016	2.38	1.75	1.78	4.23	0.000

For the time series table, first, the Dickey-Fuller test was used to measure the durability of data (*P* = 0.000), and then, the coefficients of the model were estimated using linear regression (Table 3). The results showed that there was a significant increase in the number of hospital deaths per hospital stay per day in three Kerman teaching hospitals from August 23, 2013, to September 21, 2016, during holidays. The coefficient of determination (*R*^2^) also showed that, in general, the goodness of fit of the model was appropriate but that other factors were influential in the hospital death during the holiday periods, which were not evaluated due to the lack of adequate information in the hospitals (Table 3).

**Table 3:** Time series regression of the effect of hospital variable on hospital death rate in Kerman educational hospitals from 23 August 2013 to 21 September 2016

Death rate for hospitalization per day	Hospital A		Hospital B		Hospital C	
	Coefficient	SE	Coefficient	SE	Coefficient	SE
Constant	3.26	0.26	1.68	0.16	2.20	0.27
Holiday	0.73	0.26	0.44	0.17	0.71	0.28
Goodness of fit criteria						
*R*^2^	0.006		0.006		0.005	
*F*	7.50	*P* = 0.006	6.88	*P* = 0.008	6.34	*P* = 0.012
dfuller	− 17.80	*P* = 0.000	− 18.29	*P* = 0.000	− 19.32	*P* = 0.000

**Fig. 1:**
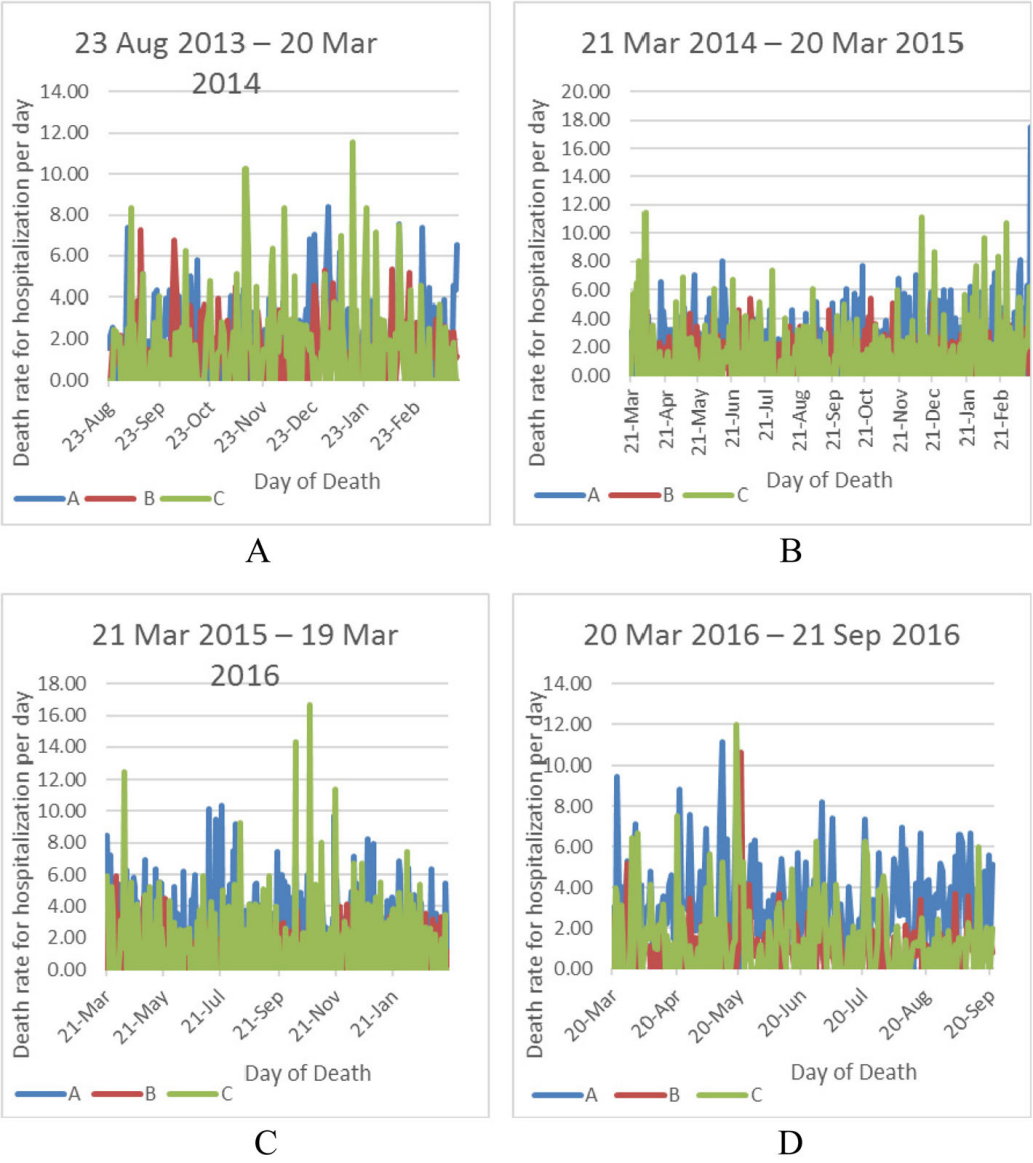
Hospital mortality time series trends for hospitalization per day in Kerman educational hospitals from 23 August 2013 to 21 September 2016. **a** Death rate from 23 Aug 2013 to 20 Mar2014; **b** Death rate from 21 Mar 2014 to 20 Mar 2015; **c** Death rate from 21 Mar 2015 to 19 Mar 2016; **d** Death rate from 20 Mar 2016 to 21 Sep 2016

A time series chart was used to better examine the time series of the days (Fig. 1). In general, the peak points of these graphs were related to the working days near to a holiday.

## Discussion

The results of this study showed that the ratio of hospital death per hospital stay per day during Nowruz holidays was 1.36 times higher than that of normal working days. Also, the most common cause of hospital deaths was due to injuries, poisoning and other consequences of external causes, while a study by Hassibi et al. showed the mortality rate due to infectious disease was higher during holidays compared to normal working days [13]. Philips et al. found that the highest mortality rate occurred during the Christmas and New Year holidays, which mainly occurred among cardiac patients [8]. According to a study, death in patients with ischemic heart disease increased over the past few years during the New Year Holidays. Also, cardiac patients’ deaths peaked between December and January, especially at the New Year’s Eve [24]. Another study showed that most deaths from cardiovascular and respiratory diseases occurred in New Year’s Eve [15], which contradicts the results of the present study. Some of the main reasons for the peak in hospital deaths caused by traffic accidents during the Nowruz holidays include increased trips, substandard roads, drowsiness, drivers’ carelessness and inexperienced drivers.

In this study, most of the hospital deaths during Nowruz holidays occurred in the morning shift. However, a study in Finland showed that most deaths occurred in the evening and night [18]. In another study, most short-term treatments during holidays took place between 8 AM and midnight [5], which contradicts the results of this study. The crowded wards, staffs’ fatigue and shortage of experienced personnel during these hours could be the reasons for the death of patients in morning shifts.

Most people who died during the Nowruz holidays were male. The study of Ahrens showed that most deaths during the New Year holidays occurred among men and were due to accidents, suicides and homicides [25]. In another study, death during Christmas and New Year holidays occurred among men more than women due to accidents and violence [26]. A study found that substance abuse and external causes (accidents, suicide and murder) were the causes of male deaths during holidays [16], which is consistent with the present study. This could be due to the greater number of men on the roads compared to women, carelessness, drowsiness, rush to reach the destination and the prevalence of riskier behaviours among males. The most deaths were in the 60–79 years age group (29.20%). A study showed that most of the dead were aged 20–39 years [16]. Another study showed that most heart patients died on vacation at the age of 20–30 years [11], which contradicted the results of this study which could be due to crashes during the Nowruz holiday, as most travels are family-related during these days, and people aged between 60 and 79 years are more vulnerable to injury.

In this study, the majority of hospital deaths during the Nowruz holidays occurred in the ICU. Similar to the results of this study, a study showed that most of the deaths during holidays occurred in the intensive care unit [18]. However, results of another study indicated that most hospital deaths occurred in the emergency department due to circulatory diseases, cancers, respiratory diseases, endocrine diseases, nutritional diseases, metabolic processes and digestive diseases [16]. A study also showed that the average number of visits to the emergency room increased by 9% during holidays, and most of these visits that occurred during holidays were unnecessary, outpatient and non-ambulance [5]. During Christmas and New Year holidays, mortality is high among patients with cardiac and non-cardiac diseases. The deaths that occurred at the time of entering the hospital in the emergency and outpatient departments peak at Christmas and New Year holidays [8]. These results contradict the findings of the present study. One of the reasons for the high number of death in the intensive care unit during holidays could be the exacerbation of patient’s condition, which is often caused by accidents, burns, consuming alcohol drinks and low quality of services.

The time series showed that, from 23 August 2013 to 20 March 2014, the highest mortality rate belonged to the hospital C and on day January 16, which could be due to road traffic accidents caused by weekend’s holiday trips. From 21 Mar 2014 to 20 Mar 2015, the highest hospital mortality rate belonged to the hospital A and on day March 20 that could be due to the New Year’s holiday, Nowruz travel and lack of full access to treatment. The highest hospital mortality rate from 21 Mar 2015 to 19 Mar 2016 belonged to the hospital C and on day March 21 that could be due to Nowruz trips, food poisoning caused by eating dishes on the roadside, burn and lack of full access to treatment. Also, the cause of the increase in hospital mortality rate on the day May 19 from 20 Mar 2016 to 21 Sep 2016 could be due to religious holidays, staff leave and lack of full access to treatment.

This study was conducted using available data and therefore faced some limitations of using existing data:

First, due to the lack of access to full personal data in hospitals, it was not possible to analyse the effects of other factors such as age, sex, hospital ward/section and cause of death in the time series. For this reason, the goodness of fit was estimated at a low level. Thus, we suggest further studies to be conducted to investigate these factors. Second, the uncertainty regarding the quality of data entry could significantly affect the results of the study.

## Conclusion

This study showed that hospital mortality to admission ratio is higher during Nowruz holidays compared to normal working days. Due to the importance of hospital mortality and its impact on the quality of services, hospital managers need to have a more accurate plan for Nowruz holidays, as in these days, hospitals are faced with the higher number of critically ill patients, shortages of staff, shortages of equipment, etc. The correct estimation of human resources, the flexibility and distribution of manpower across working shifts, the hospitals’ readiness and increased surveillance and monitoring in these days can reduce the hospital mortality.

## Data Availability

The datasets generated and analysed during the current study are not publicly available due to personal information management but are possibly available from the corresponding author on reasonable request.
